# Emerging directions for biologically active fluids

**DOI:** 10.1098/rsta.2024.0273

**Published:** 2025-09-11

**Authors:** David J. Smith, Smitha Maretvadakethope, Laurence G. Wilson, Marco Polin

**Affiliations:** ^1^ School of Mathematics, University of Birmingham, Birmingham, UK; ^2^ Life Sciences, Imperial College London, London, UK; ^3^ School of Physics, Engineering and Technology, University of York, York, UK; ^4^ Mediterranean Institute for Advanced Studies, IMEDEA, UIB-CSIC, Esporles, 07190, Spain; ^5^ Department of Physics, University of Warwick, Gibbet Hill Road, Coventry, CV4 7AL, UK

**Keywords:** fluid dynamics, biologically active fluids, active suspensions, locomotors

## 1. Introduction

*Biologically active fluids* is a broad term encompassing many crucial systems in biology and ecology. Examples include the microscopic green algae that underpin oceanic—and hence global—ecosystems; our closest unicellular relatives, the choanoflagellates; the suspensions of motile spermatozoa that contribute to sexual reproduction; and the intestinal microbiota critical to healthy physiology. Aspects of these systems have been studied by mathematicians and physical scientists for decades and indeed have provided key models for the development of theoretical tools and physical understanding in viscous fluid dynamics. Driven by advances in theoretical and experimental tools, in addition to societal motivations as diverse as climate change, biodiversity, reproductive health and clean energy, the field has undergone a rapid growth in interest and activity globally in the last 20 years. For example, the most recent American Physical Society Division of Fluid Dynamics meeting (November 2024) included 17 sessions and a plenary dedicated to the above-mentioned themes, in addition to many more sessions on higher Reynolds number biological fluid dynamics.

This special issue brings together contributions from emerging and established leaders researching the fluid dynamics of microscopic life. Phenomena of interest include the ‘continuum’ study of fluids comprising biologically active constituents—e.g. locomoting and feeding cells, microtubule suspensions, ciliated epithelia, cytoplasmic streaming—along with the microscale study of individual flagellates, ciliates and biologically inspired locomotors. While we have known the basics of how individual sperm swim and cilia propel fluid since the 1950s and 1960s, it is only more recently that the community is making significant progress in developing theories that can address the complexity of the fluid mechanics of active suspensions or the intricate interplay of swimming motions with the surrounding physical and chemical environment.

Several of the key themes for this special issue emerged from a workshop of the Biologically Active Fluids special interest group of the UK Fluids Network, held at the University of Birmingham in July 2023. Indeed the group has now held eight annual meetings and 5 years of online seminars, the latter being led by a very active and early career community with international reach. Along with the international diversity of the contributors to the present volume, it is notable that several of the contributions have early career scientists as corresponding authors.

The special issue is organized into the following three sections: (i) continuum models of active suspensions, (ii) microscale study of locomotors and their interactions with each other and their physical environment, and (iii) future directions for the study of biologically active fluids.

Section (i) is anchored by a review by Fung *et al.* of the motivation, theoretical foundations and future challenges in modelling dilute active suspensions of bacteria and algae, including pairwise and many-body interactions [1]. This review is followed by four theoretical and computational studies. Caldag & Bees assess the effect of oscillating flow on dilute suspensions and its potential for fine-tuning cell dispersion in bioreactors [2]; Ogawa & Ishimoto assess suspension-level effects of chiral properties associated with helical flagellar bundles [3]; Croze *et al.* study the impact of time-varying chemical concentration fields on bacterial chemotaxis [4]; de Graaf Sousa* et al.* describes a computational study of the effect of self-propulsion on the long-range order of densely packed active nematic fluids, which is relevant to both swarming bacteria and migrating cell layers [5]. Together these contributions exemplify the cutting edge in theoretical computational modelling of dilute and dense suspensions of swimmers, in close contact with experiments and biological/biotechnological applications.

Section (ii) brings together two reviews and four original research articles on the interaction of individual swimming cells with their physical environment. Ishikawa *et al*. review *taxis* effects on individual swimmers in response to a broad range of physical stimuli [6]; Cammann *et al.* review the breadth of active biological filaments and their mechanical behaviours and interactions across scales [7]. The first original research contribution by Kawakami & Vlahovska ([8]; see also the cover image) develops the theoretical understanding of previous observations of microswimmers in droplets, to predict the effect of the swimmer on droplet deformation and translation, as well as the coupled dynamics of the swimmer itself within the droplet. Brock *et al.* follow with an experimental investigation of the detailed three-dimensional dynamics of how bacteria and archaea with significantly different flagellar arrangements interact with surfaces, drawing out qualitatively consistent behaviours that could be exploited in rectifier devices [9]. Turning to the effects of the fluid medium itself, Gutierrez *et al.* study the response of the model flagellate *Chlamydomonas reinhardtii* to elevated fluid viscosity and explain through data analysis and simulation how and why swimming speed is reduced [10]. Finally, Neal & Bearon present a computational method for more efficient analysis of microscale propulsion in non-Newtonian fluids, demonstrating the effect of nonlinear viscoelasticity on the velocity and efficiency of a prescribed-force model swimmer [11]. Along with providing physical insights, these contributions progress the diverse tools and techniques that characterize the field: from fundamental dipolar models of swimmers, through advanced computational techniques that also utilize fundamental solutions, to experimental techniques involving digital holography, high-speed imaging and dimensionality reduction.

The final section of the special issue (iii) brings together several emerging directions for the field. Kiørboe *et al.* present an ecological perspective, challenging physical scientists to ‘harness the substantial analytical power of the physics community to address problems rooted in ecology rather than physics itself’ [12]—highlighting the diversity of species, morphologies and biological functions yet to be tackled. Moving from ecological to physiological applications, Savorana *et al.* review the field of biofilm formation, maturation and dispersal, highlighting the need to develop realistic *in vitro* models to underpin future therapeutic interventions [13]. Motivated in part by future biomedical and bioengineering applications, Severn & Lauga address the optimization of free swimming elastic filaments [14], followed by Htet & Lauga describing the development of classical resistive force theory to account for the transport of a load [15]. Both of these studies exemplify the synergy of biological applications, engineering concepts and fundamental mathematics that characterize the field. In the final article, Zheng *et al.* [16] take a somewhat different but complementary angle through with a study of diffusion-controlled growth of chemical gardens; in addition to showcasing the interplay of theory and experiment in fluid dynamics, this work is motivated by the aim to uncover the origins of the earliest living microorganisms.

Taken together, we believe that this special issue presents a good overview of the cutting edge of this field, across modelling and experimental techniques, physical insight and bio/ecological challenges.

## Data Availability

This article has no additional data.
